# microRNA-1203 targets and silences cyclophilin D to protect human endometrial cells from oxygen and glucose deprivation-re-oxygenation

**DOI:** 10.18632/aging.102795

**Published:** 2020-02-10

**Authors:** Hong-Bin Xu, Yu-Fan Zheng, Di Wu, Ya Li, Li-Na Zhou, You-Guo Chen

**Affiliations:** 1 Obstetrics and Gynecology Department, The First Affiliated Hospital of Soochow University, Suzhou, China; 2 Obstetrics and Gynecology Department, The Affiliated Changzhou No. 2 People's Hospital of Nanjing Medical University, Changzhou, China; 3 Institute of Neuroscience, Soochow University, Suzhou, China; 4 The Central Lab, North District, Suzhou Municipal Hospital Affiliated to Nanjing Medical University, Suzhou, China; 5 Department of Radiotherapy and Oncology, Affiliated Kunshan Hospital of Jiangsu University, Suzhou, China

**Keywords:** human endometrial cells, oxygen and glucose deprivation-re-oxygenation (OGDR), cyclophilin D, miR-1203, programmed necrosis

## Abstract

Oxygen and glucose deprivation (OGD)-re-oxygenation (OGDR) stimulation to the human endometrial cells mimics ischemia-reperfusion injury. Cyclophilin D (CypD)-dependent programmed necrosis pathway mediates OGDR-induced cytotoxicity to human endometrial cells. We here identified a novel CypD-targeting miRNA, microRNA-1203 (miR-1203). In T-HESC and primary human endometrial cells, ectopic overexpression of miR-1203, using a lentiviral construct, potently downregulated the CypD 3’-untranslated region (3’-UTR) activity and its expression. Both were however upregulated in endometrial cells with forced miR-1203 inhibition by its anti-sense sequence. Functional studies demonstrated that ectopic miR-1203 overexpression in endometrial cells alleviated OGDR-induced programmed necrosis, inhibiting mitochondrial CypD-p53-adenine nucleotide translocator 1 association, mitochondrial depolarization, reactive oxygen species production, and medium lactate dehydrogenase release. Contrarily OGDR-induced programmed necrosis and cytotoxicity were intensified with forced miR-1203 inhibition in endometrial cells. Significantly, ectopic miR-1203 overexpression or inhibition failed to change OGDR-induced cytotoxicity in CypD-knockout T-HESC cells. Furthermore, ectopic miR-1203 overexpression was unable to protect T-HESC endometrial cells from OGDR when CypD was restored by an UTR-depleted CypD construct. Collectively, these results show that miR-1203 targets and silences CypD to protect human endometrial cells from OGDR

## INTRODUCTION

In the clinical practices of obstetrics, postpartum hemorrhage is one common complication [1–3], causing moderate to severe ischemic injuries to human endometrium [1–3]. Endometrium ischemia is often followed by reperfusion, leading to significant oxidative injury to human endometrial cells [1–3]. At the molecular level, ischemia-reperfusion will cause reactive oxygen species (ROS) production and excessive oxidative injury to endometrial cells [4–6], leading to production of circulating lipid peroxides, but reduction of various antioxidants [4–6]. These events will cause further DNA breaks, protein damages and mitochondrial dysfunction [4–6], eventually leading to death of endometrial cells and tissues [1–3]. In experimental settings, oxygen and glucose deprivation (OGD) and subsequent re-oxygenation (OGDR) is applied to cultured endometrial cells, mimicking ischemia-reperfusion and oxidative injuries [7–10].

Our previous studies have demonstrated that OGDR primarily induced programmed necrosis (a mitochondria-dependent activate necrosis form [11–13]), but not apoptosis, in endometrial cells [14, 15]. The active necrosis pathway was evidenced by mitochondrial association of cyclophilin D (CypD)-p53-adenine nucleotide translocator 1 (ANT1), followed by mitochondrial depolarization, ROS production, and lactate dehydrogenase (LDH) release to the conditional medium [14, 15]. Importantly, CypD inhibition (by its inhibitor cyclosporine A/CsA [16]) or silencing (by targeted shRNAs) efficiently protected endometrial cells from OGDR. Reversely, ectopic overexpression of CypD intensified OGDR-induced endometrial cell necrosis [15]. Interestingly, ginseng Rh2 (GRh2), a converted ginsenoside, inhibited OGDR-induced programmed necrosis pathway and protected endometrial cells from OGDR [15]. Furthermore, activation of Akt-Nrf2 cascade by keratinocyte growth factor (KGF) inhibited OGDR-induced death in endometrial cells through shutting down the programmed necrosis pathway [14]. Therefore, our results suggest that inhibition of CypD-dependent programmed necrosis pathway is an efficient strategy to protect endometrial cells from OGDR [14, 15].

microRNAs (miRNAs) are endogenous noncoding RNAs (ncRNAs) about ~22 nucleotide (nt) long [17, 18]. miRNAs can inhibit translation and/or expression of the target mRNAs through directly binding to the 3’-untranslated region (3′-UTR) [17, 18]. One novel way to silence CypD and possibly shut down the programmed necrosis pathway is though miRNA-induced silencing of CypD. Wang et al., have shown that microRNA-30b (miR-30b) selectively silenced CypD, thus protecting heart from ischemia/reperfusion injury and necrotic cell death [19]. We here identified a novel CypD-targeting miRNA, microRNA-1203 (miR-1203). Our results further show that miR-1203 targets and silences CypD to protect human endometrial cells from OGDR-induced programmed necrosis.

## RESULTS

### miR-1203 targets and silences CypD in human endometrial cells

**Figure 1:**
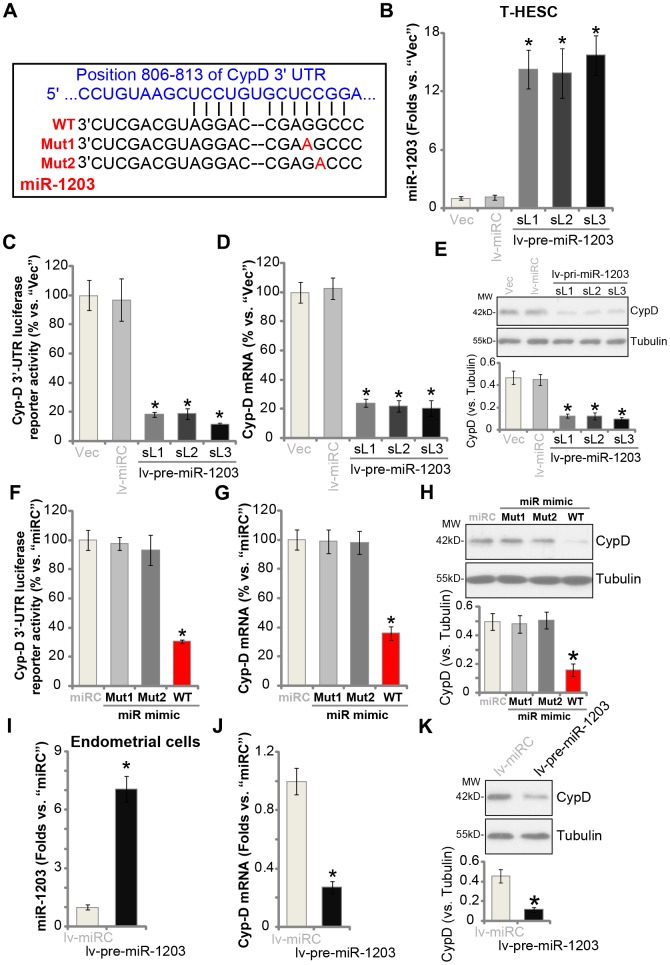
**miR-1203 targets and silences CypD in human endometrial cells.** The wild-type (WT) microRNA-1203 (miR-1203) targets CypD 3’-UTR (3’-untranslated region) at position 806-813 (**A**). T-HESC endometrial cells were infected with pre-miR-1203-encoding lentivirus (“lv-pre-miR-1203”), following puromycin selection three stable cell lines were established: “sL1/sL2/sL3”. Control T-HESC cells were infected with microRNA control lentivirus (“lv-miRC”); Expression of mature miR-1203 and CypD mRNA was tested by qPCR assays (**B** and **D**); The relative CypD 3’-UTR luciferase reporter activity was examined (**C**), with CypD protein expression tested by Western blotting assays (**E**). T-HESC cells were transfected with 500 nM of control microRNA mimic (“miRC”), the wild-type (“WT-”) or the mutant (“Mut1/2”, see sequences in **A**) miR-1203 mimics for 48h, the relative CypD 3’-UTR luciferase reporter activity (**F**), CypD mRNA (**G**) and protein (**H**) levels were tested. The primary human endometrial cells (“Endometrial cells”, same for all Figures) were infected with lv-pre-miR-1203 or lv-miRC lentivirus for 48h, expression of mature miR-1203 (**I**), *CypD mRNA* (**J**) and protein (**K**) was shown. CypD protein expression was quantified and normalized to the loading control (**E**, **H** and **K**). “MW” stands for molecular weight (same for all Figures). “Vec” stands for the empty vector control (same for all Figures). Data were presented as mean ± SD (n=5). * P <0.05 vs. “Vec”/“miRC”/“lv-miRC” cells. Experiments in this figure were repeated three times with similar results obtained.

The current study aims to silence CypD though its targeting miRNA. The possible *CypD*-targeting miRNAs, binding to its 3’-UTR, were searched from the microRNA database TargetScan (V7.2) [20]. The identified CypD-targeting miRNAs were further verified from other microRNA databases, including miRbase (v21.0) and miRDB**.** From these bioinformatic analyses we indentified that microRNA-1203 (miR-1203) putatively targets CypD’s 3′-UTR (at position 806-813, see Figure 1A), with the miR-1203-CypD 3′-UTR binding context score percentage of 99% and the context^++^ score at -0.7 (from TargetScan V7.2 [20], Figure 1A). These results indicated that there is a high possibility and specificity for the two to bind [20].

To test if miR-1203 could target and alter the expression of CypD, the pre-miR-1203-encoding lentivirus (“lv-pre-miR-1203”) was transduced to T-HESC human endometrial cells (an established human cell line) [14, 15]. Following selection by puromycin-containing complete medium, three stable cell lines were established: “sL1/sL2/sL3”. In Figure 1B qPCR results demonstrated that mature miR-1203 levels increased over 12 folds in the stable T-HESC cell lines. Importantly, the Cyp-D 3′-UTR luciferase reporter activity was largely decreased in the lv-pre-miR-1203-expressing stable T-HESC cells (Figure 1C). Furthermore, *CypD mRNA* levels reduced over 75% in the stable T-HESC cells with forced miR-1203 overexpression (*vs.* vector control cells, Figure 1D). Examining CypD protein expression, by Western blotting, confirmed that ectopic miR-1203 overexpression downregulated CypD protein expression in T-HESC cells (Figure 1E).

The results above indicated that miR-1203 selectively targets and silences CypD in T-HESC cells. To further support our hypothesis, T-HESC cells were transfected with either wild type (“WT-”) or two mutant (“Mut1/2”) miR-1203 mimics (Figure 1A). The mutants contain nucleotide mutations at the miR-1203’s binding sites to Cyp-D 3′-UTR (Figure 1A). As shown, only the WT miR-1203 mimic induced downregulation of the Cyp-D 3′-UTR luciferase reporter activity (Figure 1F) and *CypD mRNA*/protein expression (Figure 1G and 1H). While the two mutant miR-1203 mimics were completely ineffective (Figure 1F–1H). In the primary human endometrial cells, lv-pre-miR-1203 infection led to significant elevation of mature miR-1203 expression (Figure 1I), resulting in reductions in *CypD mRNA* (Figure 1J) and protein (Figure 1K) expression. The microRNA control (“miRC”) had no significant effect on miR-1203 and CypD expression in human endometrial cells (Figure 1B–1K). Collectively, these results show that miR-1203 targets and silences CypD in human endometrial cells.

### miR-1203 inhibition can elevate CypD expression in human endometrial cells

**Figure 2:**
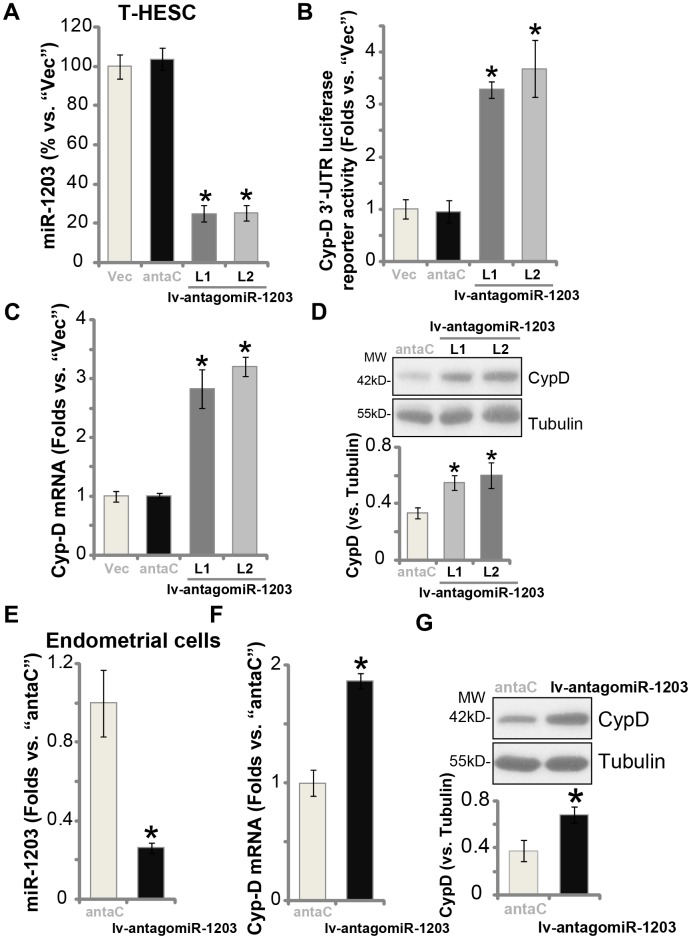
**miR-1203 inhibition can elevate CypD expression in human endometrial cells.** T-HESC endometrial cells were infected with pre-miR-1203 anti-sense lentivirus (“lv-antagomiR-1203”), following puromycin selection two stable cell lines were established: “L1/L2”. Control T-HESC cells were infected with microRNA anti-sense control lentivirus (“antaC”); Expression of mature miR-1203 and *CypD* *mRNA* was tested by qPCR assays (**A** and **C**); The relative *CypD 3’-UTR luciferase reporter activity was* examined (**B**), with CypD protein expression tested by Western blotting (**D**). The primary human endometrial cells were infected with lv-antagomiR-1203 or antaC for 48h, expression of mature miR-1203 (**E**), *CypD* *mRNA* (**F**) and protein (**G**) was shown. CypD protein expression was quantified and normalized to the loading control (**D** and **G**). Data were presented as mean ± SD (n=5), and results were normalized. * *P* <0.05 vs. “Vec”/“antaC” cells. Experiments in this figure were repeated five times with similar results obtained.

Results in Figure 1 show that miR-1203 targets and silences CypD, therefore miR-1203 inhibition could lead to CypD elevation in human endometrial cells. T-HESC cells were then infected with the lentivirus encoding the anti-sense of pre-miR-1203 (“lv-antagomiR-1203”). Puromycin was added again to establish the two stable cell lines, “L1/L2”. qPCR results, Figure 2A, show that the mature miR-1203 levels decreased over 70% in the lv-antagomiR-1203-expressing stable T-HESC cells. As a result, the Cyp-D 3′-UTR luciferase reporter activity was significantly increased (3-4 folds of control cells, Figure 2B). In T-HESC cells miR-1203 inhibition by lv-antagomiR-1203 boosted *CypD mRNA* (Figure 2C) and protein (Figure 2D) expression. Notably, the microRNA anti-sense control sequence (“antaC”) was ineffective on expression of miR-1203 (Figure 2A) and CypD (Figure 2C and 2D). In the primary human endometrial cells, lv-antagomiR-1203 infection similarly resulted in reduced expression of miR-1203 (Figure 2E), leading to increased *CypD mRNA* (Figure 2F) and protein (Figure 2G) expression (*vs.* antaC control cells). Collectively, these results show that forced miR-1203 inhibition elevated CypD expression in human endometrial cells.

### Forced miR-1203 overexpression protects human endometrial cells from OGDR-induced programmed necrosis

**Figure 3:**
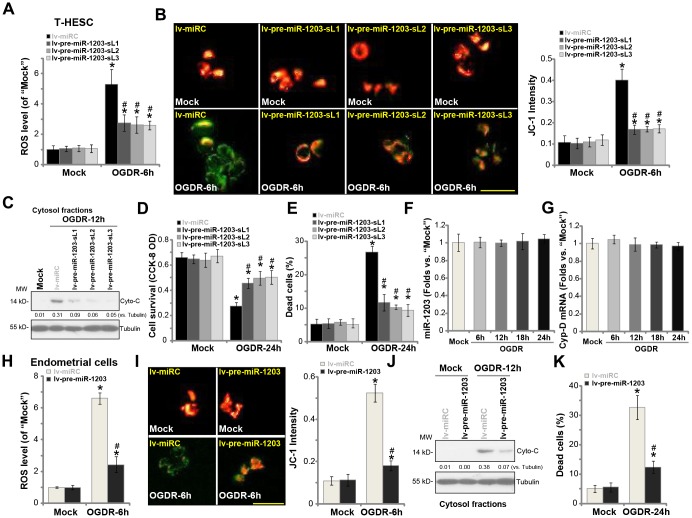
**Forced miR-1203 overexpression protects human endometrial cells from OGDR-induced programmed necrosis.** The stable T-HESC cells, with the pre-miR-1203-encoding lentivirus (“lv-pre-miR-1203-sL1/2/3”) or the control T-HESC cells with microRNA control lentivirus (“lv-miRC”), were subjected to OGD exposure for 4h, followed by re-oxygenation (“OGDR”) for applied time periods, ROS production (DCF-DA intensity, (**A**) mitochondrial depolarization (JC-1 green fluorescence accumulation, (**B**) cytochrome C release (**C**) testing cytosol proteins) were tested by the assays mentioned in the text; Cell survival and necrosis were tested by CCK-8 (**D**) and LDH release (**E**) assays, respectively. The parental control T-HESC cells were treated with the OGDR procedure for applied time periods, expression of mature miR-1203 (**F**) and *CypD* *mRNA* (**G**) was tested by qPCR assays. The primary human endometrial cells were infected with lv-pre-miR-1203 or lv-miRC lentivirus for 48h, followed by OGDR procedure for the applied time periods, ROS production (**H**), mitochondrial depolarization (**I**), cytochrome C release (**J**, testing cytosol proteins) and cell necrosis (**K**) were tested similarly. For the cytochrome C release assay, relative cytosol cytochrome C level (*vs.* Tubulin) was quantified (**C** and **J**). Data were presented as mean ± SD (n=5). “Mock” stands for non-OGDR treatment (same for all Figures). * *P* <0.05 vs. “Mock” treatment in “lv-miRC” cells. ^#^ *P* <0.05 vs. OGDR treatment in “lv-miRC” cells. Experiments in this figure were repeated three times with similar results obtained. Bar= 50 μm (**B** and **I**).

Our previous studies have demonstrated that OGDR mainly induced programmed necrosis in endometrial cells [14, 15], leading to mitochondrial CypD-p53-ANT1 association, mitochondrial depolarization, ROS production, and medium LDH release [14, 15], and eventually causing cell necrosis (but not apoptosis). On the contrary, CypD silencing or inhibition largely attenuated OGDR-induced endometrial cell necrosis [14, 15]. Since miR-1203 targets and silences CypD in human endometrial cells, we tested its activity on OGDR-induced cytotoxicity in human endometrial cells. As shown, in control T-HESC cells, OGDR stimulation induced significant ROS production (H2DCF-DA fluorescence intensity increase, Figure 3A), which was largely inhibited in the stable lines with ectopic miR-1203 overexpression (lv-pre-miR-1203-sL1/2/3, Figure 3A). Furthermore, experimental miR-1203 overexpression potently inhibited OGDR-induced mitochondrial depolarization, evidenced by JC-1 green fluorescence accumulation (Figure 3B). Additionally, the cytochrome C release to cytosol by OGDR was significantly attenuated by forced miR-1203 overexpression as well (Figure 3C). Consequently, OGDR-induced viability (CCK-8 OD) reduction (Figure 3D) and cell necrosis (medium LDH release, Figure 3E) were largely suppressed in lv-pre-miR-1203-expressing cells. These results suggest that ectopic overexpression of miR-1203 attenuated OGDR-induced programmed necrosis in T-HESC endometrial cells. Notably, the ROS scavenger N-acetylcysteine (NAC) largely ameliorated OGDR-induced cell viability reduction (Supplementary Figure 1A) and necrosis (medium LDH release, Supplementary Figure 1B) in T-HESC endometrial cells. Interestingly, OGDR treatment by itself failed to significantly change miR-1203 (Figure 3F) and CypD (Figure 3G) expression in T-HESC endometrial cells.

In the primary human endometrial cells, lv-pre-miR-1203-induced miR-1203 overexpression (see Figure 1) similarly inhibited OGDR-induced ROS production (Figure 3H), mitochondrial depolarization (Figure 3I) and cytochrome C release to cytosol (Figure 3J). Further studies demonstrated that OGDR-induced endometrial cell necrosis, or medium LDH release, was inhibited with ectopic miR-1203 overexpression (Figure 3K). These results further support that forced miR-1203 overexpression protected human endometrial cells from OGDR. Ectopic overexpression of miR-1203 had no cytotoxic effects on endometrial cells grown in normal oxygen (Figure 3A–3E, 3H–3K).

### miR-1203 inhibition can exacerbate OGDR-induced cytotoxicity in human endometrial cells

**Figure 4:**
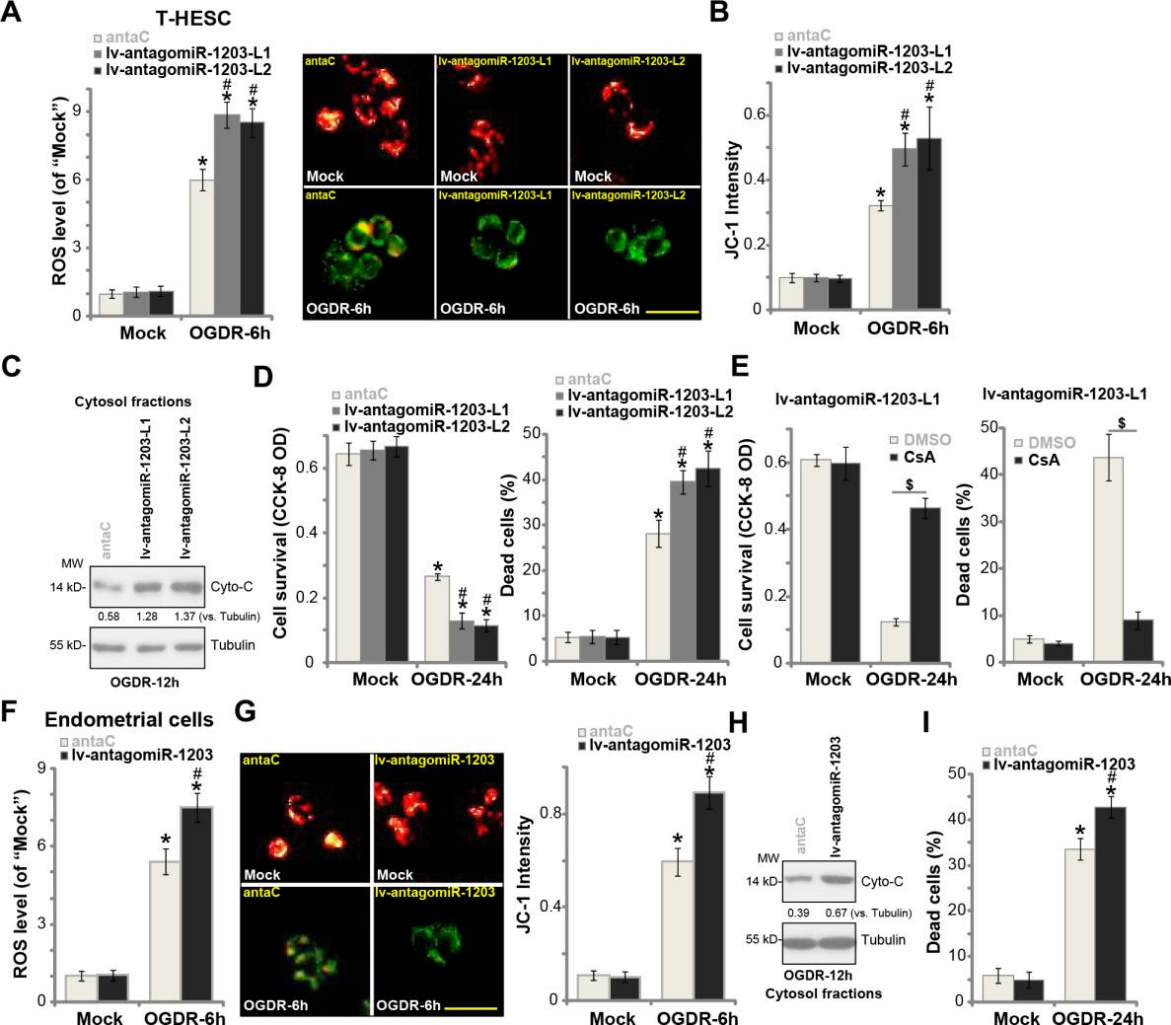
**miR-1203 inhibition can exacerbate OGDR-induced cytotoxicity in human endometrial cells**. Stable T-HESC cells with the pre-miR-1203 anti-sense lentivirus (“lv-antagomiR-1203-L1/L2”, two lines) or the microRNA anti-sense control lentivirus (“anta-C”) were subjected to OGDR for applied time periods, ROS production (DCF-DA intensity, (**A**) mitochondrial depolarization (JC-1 green fluorescence accumulation, (**B**) cytochrome C release (**C**) testing cytosol proteins) were tested by the assays mentioned in the text; Cell survival and necrosis were tested by CCK-8 and medium LDH release assays (**D**). Stable T-HESC cells with the pre-miR-1203 anti-sense lentivirus (“lv-antagomiR-1203-L1”) were pretreated with cyclosporin A (CsA, 10 μM) for 1h, followed by the OGDR stimulation for 24h, cell viability and necrosis were tested similarly (**E**). The primary human endometrial cells were infected with lv-antagomiR-1203 or anta-C lentivirus for 48h, followed by OGDR procedure for the applied time periods, ROS production (**F**), mitochondrial depolarization (**G**), cytochrome C release (**H**, testing cytosol proteins) and cell necrosis (**I**) were tested. For the cytochrome C release assay, relative cytosol cytochrome C level (*vs.* Tubulin) was quantified (**C** and **H**). Data were presented as mean ± SD (n=5). * *P* <0.05 vs. “Mock” treatment in “anta-C” cells. ^#^ *P* <0.05 vs. OGDR treatment in “anta-C” cells. ^$^ *P* <0.05 (E). Experiments in this figure were repeated five times with similar results obtained. Bar= 50 μm (**B** and **G**).

Based on the results in Figure 3, we hypothesized that experimental miR-1203 inhibition could possibly intensify OGDR-induced cytotoxicity in human endometrial cells, as it resulted in CypD upregulation (see Figure 2). The stable T-HESC cells with lv-antagomiR-1203 (see Figure 2) were subjected to OGDR procedure. As shown, as compared to control cells with anta-C (see Figure 2), lv-antagomiR-1203-expressing T-HESC cells showed increased ROS production (Figure 4A). OGDR-induced mitochondrial depolarization, or JC-1 green fluorescence accumulation, was intensified with forced miR-1203 inhibition (Figure 4B). Furthermore, after miR-1203 inhibition, cytosol cytochrome c release was significantly augmented (Figure 4C). The lv-antagomiR-1203-expressing T-HESC cells were more vulnerable to OGDR, presented with enhanced cell viability reduction (Figure 4D, the left panel) and necrosis (LDH assay, Figure 4D, the right panel), when compared to the control cells. Additional experimental results show that OGDR-induced profound cytotoxicity and necrosis in lv-antagomiR-1203-expressing T-HESC cells (“L1”) were largely attenuated by CsA, the CypD blocker [21, 22] (Figure 4E). These results indicate that forced miR-1203 inhibition induced CypD elevation, therefore aggravating OGDR’s cytotoxicity in endometrial cells.

In the primary human endometrial cells, lv-antagomiR-1203 similarly augmented OGDR-induced ROS production (Figure 4F), mitochondrial depolarization (Figure 4G) and cytochrome C release to cytosol (Figure 4H). As a result, cell necrosis was enhanced as well (Figure 4I). These results confirmed that miR-1203 inhibition exacerbated OGDR-induced cytotoxicity in human endometrial cells.

### Forced miR-1203 overexpression protects human endometrial cells from OGDR via silencing CypD

**Figure 5:**
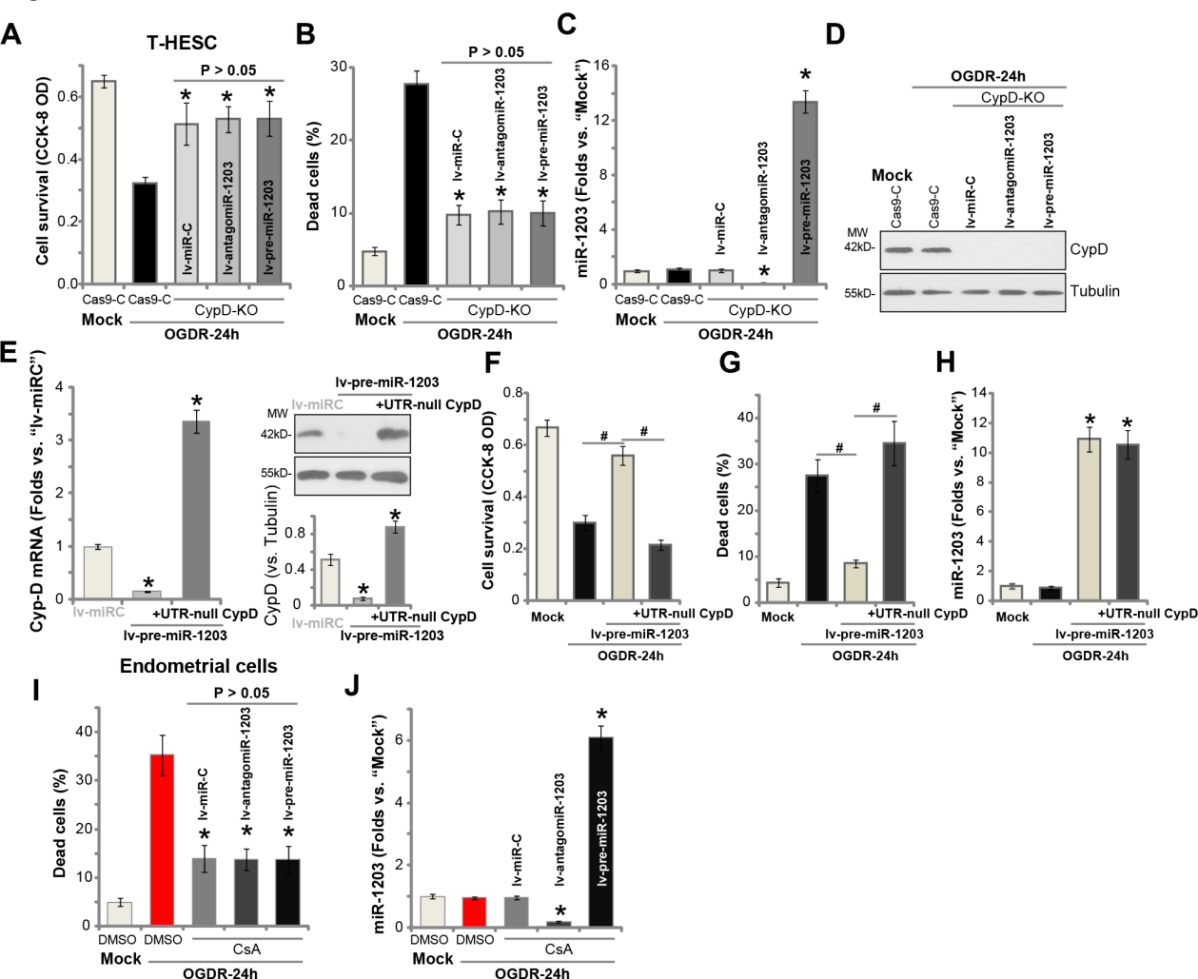
**Forced miR-1203 protects human endometrial cells from OGDR via silencing CypD.** The stable T-HESC cells with the CRISPR/Cas9-CypD-KO construct (“CypD-KO” cells) were infected with microRNA control lentivirus (“lv-miRC”), pre-miR-1203-encoding lentivirus (“lv-pre-miR-1203”), or the pre-miR-1203 anti-sense lentivirus (“lv-antagomiR-1203”), with puromycin selection the stable cells established. These cells and the CRISPR/Cas9 vector control cells (“Cas9-C”) were subjected to OGDR for 24h, cell survival and necrosis were tested by CCK-8 assay (**A**) and LDH release assay (**B**), respectively, with miR-1203 (**C**) and CypD protein (**D**) expression respectively examined by qPCR and Western blotting assays. The lv-pre-miR-1203-expression stable T-HESC cells were further transfected with or without the UTR-depleted CypD construct (“+UTR-null CypD”), after 48h *CypD mRNA* and protein expression in these cells and also in lv-miRC-expressing control cells was shown (**E**); Cells were subjected to OGDR for 24h, cell survival and necrosis were respectively tested by CCK-8 (**F**) and LDH release (**G**) assays, with miR-1203 (**H**) expression examined by qPCR. The primary human endometrial cells, with/without cyclosporin A (CsA, 10 μM) pre-treatment, were infected with lv-miRC, lv-pre-miR-1203, or lv-antagomiR-1203. After 48h cells were treated with OGDR for indicated time periods, cell necrosis and miR-1203 expression were tested by LDH (**I**) and qPCR (**J**) assays, respectively. Data were presented as mean ± SD (n=5), and results were normalized. * *P* <0.05 vs. OGDR treatment in “Cas9-C” cells (**A**–**C**). * *P* <0.05 vs. “lv-miRC” cells (**E**). ^#^ *P* <0.05 (**F** and **G**). * *P* <0.05 vs. “Mock” treatment (**H**). * *P* <0.05 vs. OGDR treatment with DMSO (vehicle control) pretreatment (**I** and **J**). Experiments in this figure were repeated four times with similar results obtained.

If miR-1203 overexpression-induced endometrial cell protection against OGDR is through silencing CypD, it should then be ineffective in the CypD-depleted cells. To test this hypothesis, we utilized the CRISPR/Cas9 method to completely knockout (KO) CypD in T-HESC endometrial cells. As shown, the CypD-KO T-HESC cells were protected from OGDR-induced cytotoxicity, showing decreased viability reduction (Figure 5A) and cell necrosis (Figure 5B) after OGDR treatment (*vs.* Cas9 vector control cells, Figure 5A and 5B). Importantly, experimentally altering miR-1203 expression, by lv-antagomiR-1203 or lv-pre-miR-1203 (Figure 5C), failed to change OGDR-induced cytotoxicity in the CypD-KO T-HESC cells (Figure 5A and 5B). Western blotting assay results, Figure 5D, confirmed CypD depletion in the CypD-KO T-HESC cells. These results show that forced miR-1203 overexpression/inhibition was ineffective against OGDR in CypD-KO cells.

To further support our hypothesis, an UTR-depleted CypD construct (“UTR-null CypD”) was transduced to miR-1203-overexpressed T-HESC endometrial cells, that completely restored *CypD mRNA* and protein expression (Figure 5E). Importantly lv-pre-miR-1203-induced T-HESC cell protection against OGDR was reversed with re-expression of the UTR-null CypD (Figure 5F and 5G). Therefore, ectopic overexpression of miR-1203 was unable to protect T-HESC cells from OGDR when CypD expression was restored (Figure 5F and 5G). Re-expression of CypD, as expected, did not change miR-1203 expression by lv-pre-miR-1203 (Figure 5H). In the primary human endometrial cells, pretreatment with CypD inhibitor CsA largely attenuated OGDR-induced cell death (Figure 5I). Importantly, experimentally altering miR-1203 expression, by lv-pre-miR-1203 or lv-antagomiR-1203 (Figure 5J), failed to change OGDR-induced cytotoxicity when CypD was blocked by CsA (Figure 5I).

## DISCUSSION

OGDR stimulation to cultured human cells mimics ischemia-reperfusion injuries [14, 15, 23–25]. Sustained OGD and following re-oxygenation will interrupt mitochondrial functions, causing significant ROS production, and profound oxidative injuries, eventually leading to cell death [14, 15, 23–25]. Interestingly, recent studies have indicated that OGDR mainly induces programmed necrosis, but not apoptosis, in human cells. For example, Liu et al., have shown that OGDR exposure to neuronal cells induced programmed necrosis, which was largely attenuated by K6PC-5, a novel sphingosine kinase 1 (SphK1) activator [25]. Wang et al., demonstrated that NKILA (NF-kappaB Interacting LncRNA) inhibition protected neuronal cells from OGDR-induced programmed necrosis [24]. Zheng et al., have shown that OGDR triggered programmed necrosis in myocardial cells, which was inhibited by ciliary neurotrophic factor (CNTF) [23].

Our previous studies have shown that CypD-dependent programmed necrosis, but not apoptosis, is the main form of cell death in endometrial cells when facing OGDR [14, 15]. CypD inhibition, by its inhibitor CsA, or shRNA-induced CypD silencing potently inhibited OGDR-induced programmed necrosis and endometrial cell death [15]. Ginsenoside Rh2 protected endometrial cells from OGDR-induced cell death by inhibiting CypD-dependent programmed necrosis pathway [15]. Conversely, forced overexpression of CypD in endometrial cells intensified OGDR-induced cell death [15]. Additionally, KGF activated Akt-Nrf2 signaling to inhibit CypD-dependent programmed necrosis pathway, thus protecting endometrial cells from OGDR [14]. Therefore targeting the CypD-dependent programmed necrosis pathway is a novel and efficient strategy to protect endometrial cells, and possible other cells [23–25], from OGDR.

miR-1203 is a relatively less-studied miRNA with unknown functions. Hu et al., have shown that circulating miR-1203 levels are downregulated in children with combined pituitary hormone deficiency (CPHD) when compared with the healthy controls [26]. Furthermore, serum miR-1203 is downregulated in certain prostate cancer patients [27]. He et al., have demonstrated that miR-1203 binds directly to Methylene Tetrahydrofolate Reductase (MTHFR) rs868014 TC or CC genotypes, resulting in their downregulation [28]. The results of this study show that miR-1203 is novel CypD-targeting miRNA. Forced overexpression of miR-1203, by lv-pre-miR-1203, decreased CypD 3′-UTR luciferase reporter activity and its expression in T-HESC cells and primary human endometrial cells. The mutant miR-1203 in the CypD 3′-UTR-binding sites failed to change CypD 3′-UTR luciferase reporter activity and expression in human endometrial cells. Conversely, CypD 3′-UTR luciferase reporter activity and expression were elavated with forced miR-1203 inhibition in endometrial cells. Therefore our results indentified miR-1203 as a novel and specific CypD-targeting miRNA in human endometrial cells.

Our studies show that miR-1203 can offer cytoprotective functions in OGDR-treated human endometrial cells. In T-HESC and primary human endometrial cells, ectopic miR-1203 overexpression attenuated OGDR-induced programmed necrosis, suppressing CypD-p53-ANT1 mitochondrial association, mitochondrial depolarization, ROS production, and medium LDH release. Conversely, OGDR-induced programmed necrosis and cytotoxicity in human endometrial cells were intensified by forced miR-1203 inhibition. Importantly, CypD silencing is absolutely required for miR-1203 overexpression-induced endometrial cell protection against OGDR. This is supported by the fact that in CypD-KO T-HESC cells ectopic miR-1203 overexpression or inhibition failed to change OGDR-induced cytotoxicity. Additionally, miR-1203 inhibition-induced aggravation on OGDR’s cytotoxicity was reversed by CypD inhibitor CsA in T-HESC cells. Furthermore, ectopic miR-1203 overexpression was unable to protect T-HESC cells from OGDR when CypD expression was restored by an UTR-depleted CypD construct. In the primary human endometrial cells, miR-1203 was also ineffective when CypD is pre-blocked by CsA.

Based on the results of this study we suggest that exogenous targeting miR-1203-CypD cascade could be a novel strategy to protect human endometrial cells from OGDR. miR-1203, and possible other CypD-targeting miRNAs, could have translational potential for the treatment of endometrium ischemia. Furthermore, considering that ischemic cardiomyopathy and ischemic stroke could share the similar CypD-dependent cell necrosis pathway in myocardiocytes [16, 19, 29, 30] and neuronal cells [31–33], Cyp-D-targeting miR-1203 might have important therapeutic value for aging patients with the two diseases.

## MATERIALS AND METHODS

### Chemical and reagents

Cyclosporine A (CsA) and puromycin were provided by Sigma-Aldrich Chemicals (St. Louis, Mo). From Gibco Co. (Suzhou, China) the cell culture reagents, including fetal bovine serum (FBS) and PBS, were purchased. The antibodies utilized in this study were obtained from Santa Cruz Biotechnology (Santa Cruz, CA) and Cell Signaling Tech (Suzhou, China). All the primers, sequences and viral constructs were designed, sequence-verified and generated by Shanghai Genechem Co. (Shanghai, China). Lipofectamine 2000 and other transfection reagents were provided by Invitrogen Thermo-Fisher (Shanghai, China).

### T-HESC cell culture

The human endometrial cell line T-HESC cells [34], from the Cell Bank of Shanghai Institute of Biological Science of CAS (Shanghai, China), were cultured under the previously-described protocol [14, 15, 34].

### Culture of primary human endometrial cells

The surgery-acquired human endometrial tissues (female, 31-year old, administrated at Changzhou Second People's Hospital, with the written-informed consent) were first digested with 0.15% trypsin-EDTA plus Collagenase I (Sigma-Aldrich) for 60 min at room temperature. The endometrial tissues were then transferred to DMEM/Hams F-12 nutrient plus FBS. Tissues were then dissolved in cold PBS and vortexed. Blood vessel cells and immune cells were abandoned using gravity sedimentation. Afterwards, the remaining primary human endometrial cells were pelleted and resuspended in the complete DMEM medium as described [15]. Primary human cells at passage 3-10 were utilized. The protocols of using human tissues and cells were approved by the Ethics Review Board of Soochow University (Suzhou, China).

### Cell viability assay

Endometrial cells were initially seeded into 96-well tissue culture plates at 5000 cells per well. The cell counting kit-8 (CCK-8) kit (Dojindo Laboratories, Kumamoto, Japan) was utilized to quantitatively measure cell viability. CCK-8 optic density (OD) was recorded at the test-wavelength of 450 nm.

### Lactate dehydrogenase (LDH) assay of cell necrosis

LDH release to the conditional medium is a quantitative measurement of cell necrosis *in vitro* [35]. A two-step LDH detection kit (Promega) was carried out to measure LDH levels in the medium, always normalized to total LDH contents [15].

### OGD/re-oxygenation (OGDR)

The OGDR procedure was described previously [14, 15, 32]. Briefly, human endometrial cells were first placed into an airtight chamber with continuous flux of gas (95% N2/5% CO2). The chamber was sealed and placed in an incubator for 4h, mimicking oxygen glucose deprivation/OGD. Cells were then returned back to the complete medium and re-oxygenated (OGDR) for applied time periods. Control cells were placed in norm-oxygenated complete medium (labeled as “Mock”).

### Western blotting

Human endometrial cells with the applied treatments were incubated with the RIPA lysis buffer with proteasome inhibitors combo (Biyuntian, Wuxi, China). The quantified protein lysate samples (40 μg per treatment in each lane) were separated by SDS-PAGE gels, transferred to PVDF blots [36]. The detailed protocol of Western blotting and data quantification (using the ImageJ software) were described in detail in our studies [14, 15].

### Mitochondrial depolarization

In the stressed cells with mitochondrial depolarization (“ΔΨ”) the red JC-1 dye shall aggregate in mitochondria to form green monomers [37]. Following the applied treatments human endometrial cells were incubated with JC-1 (5 μg/mL) for 15 min (under the dark). JC-1 green fluorescence intensity was measured under a fluorescence spectrofluorometer at 530 nm (Titertek Fluoroscan, Germany). The representative JC-1 images, integrating both green and red fluorescence images, were also presented.

### ROS detection

As reported early [14, 15], the fluorescent dye DCFH-DA (2′,7′-dichlorofluorescein diacetate) assay was applied to examine ROS levels [38–40]. The human endometrial cells were initially seeded into 96-well tissue culture plates at 5000 cells per well. Following the applied treatments, cells were incubated with DCFH-DA (50 μM) for 30 min. The DCF fluorescence, reflecting cellular ROS intensity, was detected by the above-described fluorescence reader.

### Quantitative real-time PCR (qPCR)

After treatment, cellular RNA was extracted and complementary DNA (cDNA) was synthesized as described [14, 15]. We utilized the the ABI Prism 7600 Fast Real-Time PCR system for qPCR assay. The product melting temperatures were calculated by the melt curve analyses. Glyceraldehyde-3-phosphatedehydrogenase (GAPDH) mRNA was tested as the reference gene and the internal control, using the 2−ΔΔCt method for quantification. The mRNA primers of human CypD and GAPDH were described previously [41]. miR-1203 was normalized to U6. miR-1203 and U6 primers were obtained from OriGene (Beijing, China).

### Forced overexpression or inhibition of miR-1203

The pre-miR-1203 nucleotide sequence (UCCUCCCCGGAGCCAGGAUGCAGCUCAAGCCACAGCAGGGUGUUUAGCGCUCUUCAGUGGCUCCAGAUUGUGGCGCUGGUGCAGG) and the anti-sense sequence (UGCUGUGGCUUGAGCUGCAUCCUGGCUCCGGGGAG) were synthesized and verified by Shanghai Genechem Co. (Shanghai, China). Each was inserted into a GV248 lentiviral construct (Shanghai Genechem Co.). The construct and the lentivirus-packing plasmids (psPAX2 and pMD2.G, from Dr. Jiang [42]) were transfected together to HEK-293T cells, establishing pre-miR-1203-expressing lentivirus (“lv-pre-miR-1203”) or pre-miR-1203 anti-sense lentivirus (“lv-antagomiR-1203”). Viruses were enriched, filtered, and added to cultured human endometrial cells, and cultured in the polybrene-containing complete medium. For T-HESC cells puromycin (5.0 μg/mL) was added to select the stable cells. The mature miR-1203 (with sequence CCCGGAGCCAGGAUGCAGCUC) levels were always tested by qPCR.

### The assaying of CypD 3'-UTR luciferase reporter activity

The CypD 3'-UTR reporter plasmid (pMIR-REPORT plasmid, containing the miR-1203-binding sites, at position 806-813, generated by Shanghai Genechem Co) was transfected to endometrial cells by Lipofectamine 2000. Afterwards, cells were subjected to the applied genetic manipulations, CypD 3'-UTR luciferase activities examined by a Promega kit [43].

### Transfection of miR-1203 mimic

T-HESC cells were seeded onto the six-well tissue-culturing plates (1 × 10^5^ cells/well). Transfection of 500 nM of the wild-type (“WT-”) or the two mutant (“Mut-”) miR-1203 mimics (both provided by Shanghai Genechem Co.) was carried out by the Lipofectamine 2000 protocol for 48h.

### CypD over-expression

The CypD pSuper-puro-Flag vector, without 3’-UTR, was reported early [44]. The CypD construct was transfected to T-HESC cells through Lipofectamine 2000. After 24h, cells were selected by puromycin (5.0 μg/mL) for another 2 days. CypD overexpression in the resulting cells was verified by qPCR and Western blotting assays.

### CypD KO

At 1×10^5^ cells per well T-HESC cells were seeded into 6-well plates. The small guide RNA (sgRNA) targeting human Nrf2 (targeted DNA sequence, GGCGACTTCACCAACCACAA) was inserted into the lentiCRISPR-GFP plasmid (provided by Dr. Cao [45]). The plasmid was transfected to T-HESC cells. After 48h, the transfected cells were subjected to GFP-sorting, and single cell line was established. GFP KO in the stable cells was verified by qPCR and Western blotting assays.

### Statistical analysis

Data in this study were presented as mean ± standard deviation (SD). Repeated-measures analysis of variance (RMANOVA) followed by Dunnett’s post hoc test for multiple comparisons (SPSS 16.0) were applied to evaluate statistical significance of observed differences, using SPSS21.0 (SPSS Co. Chicago, CA). To determine significance between two treatment groups, the two-tailed t tests were carried out (Excel 2007). Significance was chosen as *P* < 0.05.

## Supplementary Material

Supplementary Figure 1
