# Supplementary material for: microRNA-1203 targets and silences cyclophilin D to protect human endometrial cells from oxygen and glucose deprivation-re-oxygenation
Source: Aging (Albany NY). 2020 Feb 10;12(3):3010–24. doi: 10.18632/aging.102795 (PMC7041737; doi:10.18632/aging.102795)
Supplement: Supplementary Figure 1 [file aging-12-102795-s001..pdf]

SUPPLEMENTARY FIGURE

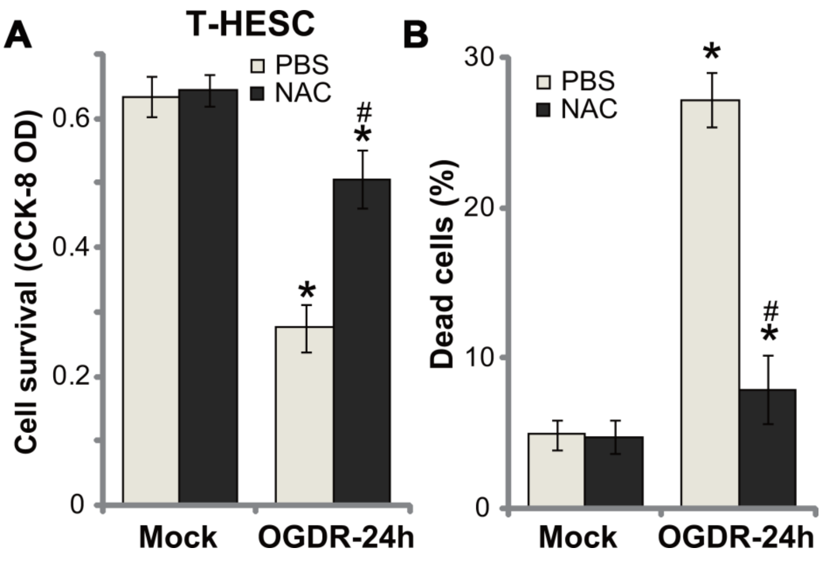

Supplementary Figure 1. T-HESC cells were pretreated with anti-oxidant N-acetylcysteine (NAC, 500 μM) for 1h, followed by OGDR stimulation for 24h, cell survival and necrosis were tested by CCK-8 (**A**) and LDH release (**B**) assays, respectively. Data were presented as mean ± SD (n=5).* *P* <0.05 *vs.* PBS pretreatment in "Mock" cells. # *P* <0.05 *vs.* PBS pretreatment in "OGDR" cells. Experiments in this figure were repeated three times with similar results obtained.
